# Implications of possible interpretations of ‘greenhouse gas balance’ in the Paris Agreement

**DOI:** 10.1098/rsta.2016.0445

**Published:** 2018-04-02

**Authors:** J. Fuglestvedt, J. Rogelj, R. J. Millar, M. Allen, O. Boucher, M. Cain, P. M. Forster, E. Kriegler, D. Shindell

**Affiliations:** 1 CICERO Center for International Climate Research, PO Box 1129, Blindern, 0318 Oslo, Norway; 2 Energy Program, International Institute for Applied Systems Analysis (IIASA), 2361 Laxenburg, Austria; 3 Institute for Atmospheric and Climate Science, ETH Zurich, Universitätstrasse 16, 8006 Zurich, Switzerland; 4 Environmental Change Institute, School of Geography and the Environment, University of Oxford, South Parks Road, Oxford OX1 3QY, UK; 5 Department of Physics, University of Oxford, Parks Road, Oxford OX1 3PU, UK; 6 Institut Pierre-Simon Laplace, Sorbonne Université, CNRS, Paris, France; 7 Oxford Martin School, University of Oxford, 34 Broad Street, Oxford OX1 3BD, UK; 8 School of Earth and Environment, Maths/Earth and Environment Building, University of Leeds, Leeds LS2 9JT, UK; 9 Potsdam Institute for Climate Impact Research (PIK), Member of the Leibniz Association, PO Box 601203, 14412 Potsdam, Germany; 10 Nicholas School of the Environment, Duke University, Durham, NC 27708, USA

**Keywords:** Paris Agreement, net-zero emissions, CO_2_ equivalence, greenhouse gas balance, emission metrics

## Abstract

The main goal of the Paris Agreement as stated in Article 2 is ‘holding the increase in the global average temperature to well below 2°C above pre-industrial levels and pursuing efforts to limit the temperature increase to 1.5°C’. Article 4 points to this long-term goal and the need to achieve ‘balance between anthropogenic emissions by sources and removals by sinks of greenhouse gases'. This statement on ‘greenhouse gas balance’ is subject to interpretation, and clarifications are needed to make it operational for national and international climate policies. We study possible interpretations from a scientific perspective and analyse their climatic implications. We clarify how the implications for individual gases depend on the metrics used to relate them. We show that the way in which balance is interpreted, achieved and maintained influences temperature outcomes. Achieving and maintaining net-zero CO_2_-equivalent emissions conventionally calculated using GWP_100_ (100-year global warming potential) and including substantial positive contributions from short-lived climate-forcing agents such as methane would result in a sustained decline in global temperature. A modified approach to the use of GWP_100_ (that equates constant emissions of short-lived climate forcers with zero sustained emission of CO_2_) results in global temperatures remaining approximately constant once net-zero CO_2_-equivalent emissions are achieved and maintained. Our paper provides policymakers with an overview of issues and choices that are important to determine which approach is most appropriate in the context of the Paris Agreement.

This article is part of the theme issue ‘The Paris Agreement: understanding the physical and social challenges for a warming world of 1.5°C above pre-industrial levels'.

## 1. Introduction

**Figure 1.**
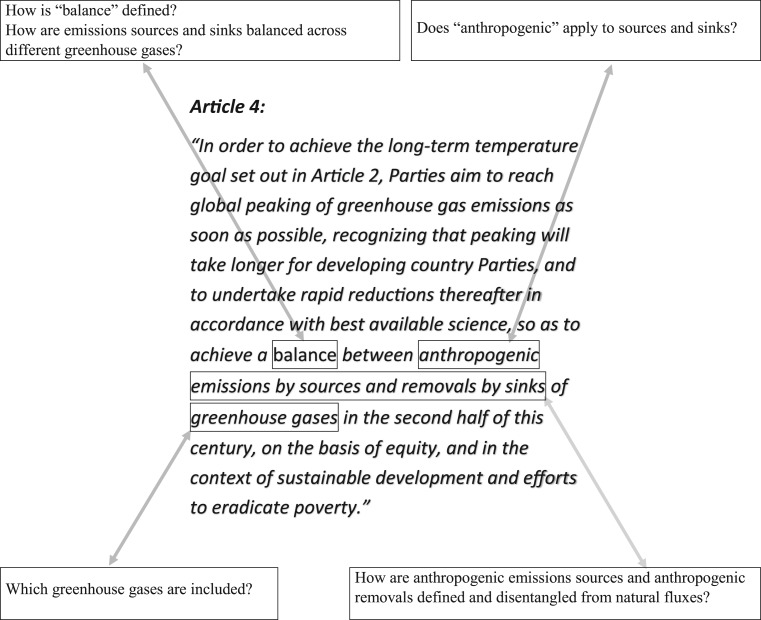
Elements of Article 4 discussed in this analysis.

The Paris Agreement was adopted by 195 countries in December 2015, putting in place a legal architecture for international climate action. On 4 November 2016, the agreement entered into force. Article 2 of the agreement contains the formulation of the main goal: ‘holding the increase in the global average temperature to well below 2°C above pre-industrial levels and pursuing efforts to limit the temperature increase to 1.5°C above pre-industrial levels’. In the context of this long-term temperature goal, Article 4 sets a greenhouse gas (GHG) mitigation goal of achieving ‘a balance between anthropogenic emissions by sources and removals by sinks of greenhouse gases'. Earlier studies have discussed various related concepts such as carbon neutrality, climate neutrality and net-zero carbon or net-zero GHG emissions (e.g. [1–5]). Here, we explore how the GHG balance objective of Article 4 can be interpreted from a scientific perspective and analyse the scientific implications of possible policy choices. Figure 1 gives a selective overview of various elements of Article 4 that may be subject to interpretation and which related questions may be raised.

## 2. Indicator and level of balance

The word ‘balance’ alludes to an equilibrium concept where, for example, flows of GHGs into and out of the atmosphere are balanced to minimize further anthropogenic perturbation of the climate system. Because there are several anthropogenic GHGs, which are subject to both anthropogenic influences and natural variability, a question that follows is what type of balance or equilibrium is aimed at. We here describe several possible ‘balance’ interpretations in terms of particular climatic quantities, with varying degree of consistency with the text of the Paris Agreement and the UNFCCC [6].

The main goal of the Paris Agreement is formulated in terms of change in the *global mean temperature* relative to pre-industrial levels. This indicator can, therefore, guide the interpretation of reaching a balance between sources and sinks. The balanced *net* emissions (i.e. the difference between emissions and removals) could thus in one interpretation correspond to those GHG emissions that *stabilize the global mean temperature* at some given level (within natural climate variability). Note, however, that the Paris Agreement makes no reference to whether the global mean temperature is to be stabilized at some level above pre-industrial levels or whether it has to peak and thereafter decline again. As the Paris Agreement's long-term temperature goal does not put an end date on the target to ‘pursuing’ limiting global temperature rise to 1.5°C, a trajectory of the global temperature peaking and declining is thus not excluded.

A second hypothetical interpretation of ‘balance’ could be evaluated in terms of the GHG emissions that stabilize radiative forcing (RF) at some level. These two interpretations differ because the climate system is currently out of radiative balance, but become equivalent once climate equilibrium is reached.

A third, and related, definition of balance could be the point at which ‘CO_2_-forcing-equivalent’ [7] emissions reach zero, where ‘CO_2_-forcing-equivalent’ is defined as the CO_2_ emissions that give the same total and rate of change of RF for all components combined as CO_2_ alone. Net-zero CO_2_-forcing-equivalent (CO_2_-fe) emissions lead to a gradually declining RF, just as RF declines slowly after anthropogenic emissions reach zero in the case in which CO_2_ is the only radiatively active agent. As CO_2_-fe emissions behave, by construction, exactly like CO_2_, it follows that the well-known result that net-zero CO_2_ emissions correspond to zero CO_2_-induced warming [8] also applies. Hence, sustained net-zero CO_2_-fe emissions represent a better predictor of global temperature stabilization than stable atmospheric composition or RF (see the second interpretation, above), which is associated with a substantial residual long-term warming commitment.

A fourth interpretation, which will be explored in later sections, is that the ‘balance’ can be interpreted in terms of net-zero *CO_2_-equivalent emissions*. This requires a decision on how to calculate CO_2_-equivalent emissions by weighting different GHGs. In particular, the meaning of net-zero GHG emissions varies with the chosen emission metric, and so does the climate effect of maintaining this ‘balance’ over time.

The Paris Agreement text is not explicit about whether the balance between sources and sinks is mandated at a global or national level. From a climate system perspective, the global level matters most. However, there are significant differences in anticipated residual (i.e. costly and hard to abate) emissions across countries; some countries have high emissions from agriculture, heavy industry, etc., that may be costly or difficult to bring to zero. Furthermore, emissions from international aviation and maritime transport are currently not attributed to individual countries. There are also differences in the potentials for net negative CO_2_ emissions across countries due to differences in forest volume and characteristics, biofuels and carbon-free renewables, sequestration capacity, etc. Thus, even in an ambitious low-emission world, the achievement of balance at the national level will be differentiated and diverse in space and time. To achieve net-zero emissions at a global level, mechanisms will be needed for strong international coordination. Various mechanisms (e.g. emission trading) could be elements of an international regime that aims to obtain balance at a global scale.

Several unresolved scientific and political issues remain related to active carbon dioxide removal (CDR) from the atmosphere (e.g. [9]) and many of these also apply to the context of GHG balance, such as who should pay for the implementation and maintenance of CDR measures that are needed to balance emissions from various activities within a country or across countries? Several questions related to responsibilities and coordination need to be considered from various perspectives and disciplines [10].

Solar radiation management (SRM) by injecting sulfate aerosols into the atmosphere has also been suggested as a measure to limit warming (e.g. [11–13]). However, such a response strategy is not covered by the text of the Paris Agreement and can be excluded from considerations of a GHG balance.

## 3. Does ‘anthropogenic’ apply to emission sources and removal sinks?

Determining whether the word ‘anthropogenic’ in Article 4 applies to both emission sources and removals by sinks is essential to quantify the implications of the Paris Agreement's ‘balance’ (figure 1).

The combined information available in the Paris Agreement indicates that only anthropogenic sources and anthropogenic sinks are to be considered to determine the Agreement's ‘balance’. Article 29 of the Paris Agreement states that the Arabic, Chinese, English, French, Russian and Spanish texts of the Agreement are all equally authentic. Both the Spanish and the French versions of the Agreement state explicitly that the required balance applies to anthropogenic emissions and anthropogenic removals^1^. Many articles in international agreements are necessarily left open to interpretation, and subtle differences between translations are difficult to avoid. In this case, however, we assume that the more precise wording as spelled out literally in the French and Spanish versions of the Agreement removes most of the ambiguity present in the other language versions regarding the application of ‘anthropogenic’.

An additional comparison of Article 4 with other documents under the United Nations Framework Convention on Climate Change (UNFCCC) seems to further support the exclusion of natural sinks. Specifically, the wording of Article 4 is similar to the earlier use of the phrase ‘anthropogenic emissions by sources and removals by sinks’ in the original UNFCCC text (Article 4.1(a) in [6]), which stipulates that Parties shall: ‘Develop, periodically update, publish and make available to the Conference of the Parties, […] national inventories of anthropogenic emissions by sources and removals by sinks of all greenhouse gases not controlled by the Montreal Protocol, using comparable methodologies to be agreed upon by the Conference of the Parties’. The Paris Agreement Article 4 language thus directly mirrors this earlier UNFCCC language, and in order to determine how ‘anthropogenic emissions by sources and removals by sinks of all greenhouse gases’ is understood under the UNFCCC, one can hence look at existing reporting rules developed for the UNFCCC national inventories. This understanding is further supported by clarifications in the Paris Agreement. For instance, although the reporting rules for anthropogenic GHG emissions and removals have not yet been decided, the Paris Agreement already stipulates that ‘[i]n the context of their nationally determined contributions, when recognizing and implementing mitigation actions with respect to anthropogenic emissions and removals, Parties should take into account, as appropriate, existing methods and guidance under the Convention’. These various lines of evidence thus suggest that the GHG balance described in the Paris Agreement refers to achieving net-zero anthropogenic GHG emissions [3], although details are still to be clarified and decided upon.

## 4. How are ‘anthropogenic emissions by sources and removals by sinks' defined?

The definition of *anthropogenic emissions* of GHGs is relatively intuitive and reporting guidelines exist in the UNFCCC for emissions related to domestic, industrial, agricultural and forestry activities. The definition of *anthropogenic removals* is more subject to debate, and previous interpretations and definitions have to be reassessed in the light of the Paris Agreement, new emerging technologies and the body of scientific literature on CDR or ‘negative emissions’ (e.g. [14]).

The Intergovernmental Panel on Climate Change (IPCC) provides Reporting Guidelines for GHG emissions and removals in the context of the UNFCCC [15]. From these guidelines, it is clear that anthropogenic removals refer to the removal of CO_2_ from the atmosphere by a sink due to human activities. Currently, this is particularly the case in the land-use, land-use change and forestry (LULUCF) sector. The UNFCCC already offers the possibility for emissions inventories to include CO_2_ removal associated with carbon sequestration from afforestation, agricultural practices and, more generally, the management of terrestrial ecosystems, and a number of Annex 1 countries routinely report carbon forest sinks. These reported sinks are not fully anthropogenic in that indirect effects (e.g. due to fertilization by atmospheric CO_2_ and/or nitrogen deposition) and natural effects on managed land are also included. The exact definition of land-use emissions is a challenge, and may vary between land-use modelling and integrated assessment models (IAMs) on the one hand, and estimates from UNFCCC accounting rules on the other. There is also no sophisticated treatment of land-use management in the current generation of Earth system models yet. As a result, so-called permissible CO_2_ emissions associated with a given concentration or temperature pathway may not be directly comparable to the net global CO_2_ emissions as per UNFCCC reporting rules when there is a significant contribution from CO_2_ removals by ecosystems [16]. Finally, natural sinks can persist up to several decades after CO_2_ concentrations have peaked in 1.5°C or 2°C mitigation pathways [17] due to the fact that the carbon cycle is currently out of equilibrium.

The prospect of novel CDR techniques can raise new and different sets of questions. CDR technologies can be broadly grouped according to where they store the atmospheric CO_2_. Besides the terrestrial CDR measures in the LULUCF sector, there exist geological CDR techniques like bioenergy combined with carbon capture and storage (BECCS), direct air capture and storage, and enhanced weathering, as well as ocean-based CDR measures like ocean alkalinization and ocean iron fertilization. Geological CDR sequesters the CO_2_ in geological formations or mineralizes it. BECCS, in particular, has been receiving increasing attention in recent years. Owing to its reliance on bioenergy, it can impact anthropogenic emissions and removals in the LULUCF sector that require robust reporting. However, its CDR component, the sequestration of CO_2_ in geological formations, occurs in the energy sector and would need to be accounted for in terms of how much of the biomass carbon is transferred to geological storage (avoiding double counting with LULUCF fluxes). Ocean-based CDR measures would raise a host of additional questions concerning the governance of international waters, including the London Convention on the Prevention of Marine Pollution, and the reporting of territorial emissions. And when a natural sink like the oceans or the terrestrial biosphere is enhanced by some anthropogenic action or by climate change itself, it may be a matter of interpretation how much of it should be considered anthropogenic.

Despite this complexity, there are some basic properties of the carbon cycle that allow assessment of the permanence of different storage options. Carbon is exchanged between atmospheric, oceanic and biospheric reservoirs, such that, for example, the rate of increase in atmospheric load is only about half present-day net CO_2_ emissions. Uptake of the remainder by the terrestrial biosphere and oceans is often referred to as a ‘natural sink’, but it represents a redistribution of carbon between finite-capacity reservoirs, not permanent removal. The additional carbon taken up by the oceans and biosphere may continue to play a role in the climate system even though it is not present in the atmosphere because, for example, it would partially be released again if atmospheric CO_2_ levels were to be artificially drawn down by CDR. Thus, the time scales and reversibility associated with enhanced CO_2_ uptake by terrestrial and ocean-based CDR measures are critical.

By contrast, natural geological processes that remove carbon from the active carbon cycle and resequester it into the lithosphere are so slow relative to anthropogenic sources and sinks that such removals, although permanent, can be largely ignored on multi-century time scales. Hence, in a strict sense, only sequestration of CO_2_ in geological formations with negligible leakage or enhanced weathering and artificial remineralization techniques represent permanent sinks to balance the extraction of fossil carbon.

Thus, an important issue is the *permanence* of the CO_2_ removal process under consideration. This applies to both industrial and ecosystem-based CO_2_ removal processes [18]. Carbon sequestered in soils and ecosystems may return to the atmosphere as CO_2_ on time scales of years to decades in the case of a change in agricultural practices or timber harvest. Forest fires are another example by which a ‘natural’ process may cancel the effect of land management practices favouring carbon sequestration. It can be argued that uptake of CO_2_ by the biosphere might only count as a ‘removal’ in the context of Article 4 if it could be guaranteed that the relevant CO_2_ will not be re-released into the atmosphere over time scales of centuries; or alternatively, if emissions inventories would also confidently include potential re-release of carbon from such temporary reservoirs and count it towards anthropogenic emissions. In the case of carbon capture and storage or BECCS, a fraction of the carbon sequestered in geological reservoirs may also leak back to the atmosphere as CO_2_. As a result, 1 kg of CO_2_ emitted at a given time may not be equivalent to 1 kg of CO_2_ removed at the same time. Kim *et al.* [18] have shown that CO_2_ removals through land-based carbon sequestration may have to be discounted by up to 50% to account for the impermanence of the storage or the existence of maintenance costs. While the latter study [18] focused on agricultural tillage and forest management practices, the argument holds for other carbon sequestration techniques if there is a risk for carbon leakage or if there are maintenance or monitoring costs. This issue has received little echo so far in policymaking but could be a way to address the issue of permanence of the sinks.

For short-lived climate forcers (SLCFs) such as methane, the interpretation of ‘removals by sinks’ also requires clarification in the context of the UNFCCC. Approximately 10% of atmospheric methane is oxidized every year, so a steady rate of anthropogenic methane emissions maintains the atmospheric methane load above its natural level by an amount approximately 10 times the annual rate of anthropogenic methane emissions. If anthropogenic emissions were to cease, methane concentrations would revert to a level in balance with natural sources within a few decades. Importantly, however, unlike the uptake of CO_2_ in oceans and biosphere, the removal of methane through oxidation is permanent and irreversible (but it produces CO_2_—which is a net addition to atmospheric burden if the CH_4_ is of fossil origin).

Enhanced concentration of an atmospheric species generally increases its removal rate. Taking methane as an example, it may be argued that the enhanced rate of oxidation due to anthropogenic elevation of atmospheric CH_4_ concentrations constitutes an ‘anthropogenic removal’. Under these assumptions, a constant rate of anthropogenic emissions that stabilizes atmospheric methane concentrations may be considered as achieving a ‘balance’ between anthropogenic sources and anthropogenic sinks. On the one hand, oxidation is a natural process whose rate has been increased by the anthropogenic elevation of atmospheric CH_4_ concentrations; on the other hand, this additional methane oxidation would not be happening in the absence of human influence on climate, because the additional methane would not be there. This can be seen in the light of the IPCC emission reporting guidelines, which only consider emissions removals if they occur on *managed* land. Despite methane being included in emissions inventories since the very beginning, in no instance do IPCC guidelines suggest that atmospheric oxidation processes would be counted towards anthropogenic removals.

Active direct anthropogenic removals of non-CO_2_ gases such as CH_4_ and N_2_O are in principle possible [19,20] but are much more speculative. In the case of catalytic destruction of these gases, there is no issue of permanence of the removal, but the climate effects of by-products have to be accounted for before setting an equivalence between emissions and removals.

## 5. Which drivers of climate change are included?

Human activities affect climate through emissions of gases, aerosols and changes in land surface characteristics (e.g. [21]) and the Intended Nationally Determined Contributions (INDCs), submitted prior to COP21, include a broad set of components beyond well-mixed GHGs. For example, some countries included measures to reduce short-lived forcers such as nitrogen oxides (NOx), carbon monoxide (CO) and black carbon (BC) aerosols. However, the words ‘greenhouse gases’ in Article 4 exclude aerosols and aerosol precursor gases (e.g. BC and SO_2_) from the group of components included in the evaluation of ‘balance’. By convention, this phrase also probably implies well-mixed GHGs, excluding tropospheric and stratospheric ozone changes. In the case of methane, however, indirect short-lived GHG responses are included. That is, the tropospheric ozone chemical response is routinely included (via the global warming potential for CH_4_), creating a situation where one ozone precursor's impact on climate via tropospheric ozone is accounted for, whereas all others are not. Changes in stratospheric ozone and water vapour and the climate impacts of these are also generally included for methane.

The Kyoto Protocol adopted a basket of six gases or groups of gases: CO_2_, CH_4_, N_2_O, SF_6_, perfluorocarbons (PFCs) and hydrofluorocarbons (HFCs)—and it may be argued that this is the group of gases that could by default be included in the ‘greenhouse gas balance’. (Later, NF_3_ was added to the basket of ‘Kyoto II’.) The GHG chlorofluorocarbons (CFCs) and hydrochlorofluorocarbons (HCFCs) are regulated under the Montreal Protocol, and HFCs are now also likely to be regulated under the Montreal Protocol through the Kigali Amendment.

## 6. Emission metrics for calculation of ‘CO_2_ equivalence’

Emission metrics have traditionally been used in policymaking and climate change assessments for the calculation of ‘equivalent’ amounts of non-CO_2_ and CO_2_ emissions. The global warming potential (GWP), introduced by the IPCC in 1990, is the most widely used metric to calculate this equivalence. It represents the time-integrated RF from a pulse emission of one mass unit of a GHG or other radiatively active species over a chosen time horizon following its emission, relative to the corresponding impact of the emission of one mass unit of CO_2_. Multiplying an emission of gas *i* by its GWP, for a chosen time horizon, gives the so-called ‘CO_2_-equivalent emission’. Ideally, equivalence should give the same climate impact in a given physical climate variable of interest, and for this emission metric it gives equivalence in terms of integrated RF over the chosen time horizon. The GWP has been subject to criticism (e.g. [22]) and regular re-evaluation (e.g. [21]). A new application of GWP, denoted here as GWP*, is designed to compare the impact of climate-forcing agents with very different lifetimes on future temperatures [5]. It has been shown that conventional GWP values can be used to approximate the relative impact of both long-lived (cumulative) GHGs and SLCFs on global temperatures, requiring only a change in usage, but no new metric values or concepts. This new way of using the traditional GWP metric considers the temperature change due to a sustained increase of one tonne per year in the emission rate of a short-lived component (as proposed in [23]) to be approximately equivalent to the temperature change due to a one-off pulse emission of GWP*_H_* *×* *H* tonnes of CO_2_, where GWP*_H_* is the value of that short-lived component GWP for a time horizon, *H* [5]. Short- and long-lived components are here distinguished by having characteristic atmospheric residence times less than or greater than *H,* respectively.

Various alternative metrics have been proposed in the literature, and among these, the global temperature change potential (GTP) [23,24] has so far received most attention. The GTP represents the impact of the emission of one mass unit of a GHG on global temperature at a specified point in time after emission, again relative to the corresponding impact of the emission of one mass unit of CO_2_. (Both GWP and GTP include natural sinks in their formulations.)

The INDCs submitted ahead of COP21 referred to different emission metrics—mainly GWPs from various IPCC assessments (Second Assessment Report (SAR), the 3rd Assessment Report (TAR), the 4th Assessment Report (AR4) and the 5th Assessment Report (AR5)), and in a few cases GTP. The text of the Paris Agreement leaves the choice of metric open, and paragraph 31 of the Paris outcome (Decision 1/CP.21) requests that Parties will report emissions and removals using common metrics assessed by the IPCC and adopted by the Conference of the Parties serving as the meeting of the Parties to the Paris Agreement.

Another candidate for calculating CO_2_ equivalence is expressing GHGs in terms of ‘CO_2_-forcing-equivalent emissions' (CO_2_-fe). The temperature response to aggregate CO_2_-fe emissions is, by construction, identical to the response to the equivalent emissions of CO_2_ only. Net-zero CO_2_-fe emissions thus correspond to gradually declining RF and stable temperatures [8]. Diagnosing CO_2_-fe emissions requires a carbon cycle model, but a simple approximation can also be used to estimate CO_2_-fe emissions as they approach zero. Because the forcing integrated over a time horizon *H* resulting from a pulse injection of CO_2_ increases approximately linearly with *H* over the range of values of *H* typically used in policy calculations (as shown in fig. 8.29 of [21]), the rate of CO_2_-fe emissions is given, to a good approximation, by the rate of change of RF multiplied by *H*/AGWP*_H_*(CO_2_) [23], where AGWP*_H_*(CO_2_) is the absolute GWP of CO_2_ for time horizon *H* [21]. Hence, to a good approximation, net-zero CO_2_-fe emissions corresponds to a combination of residual (positive or negative) CO_2_ emissions balancing a low rate of change of non-CO_2_ RF given by 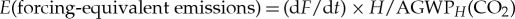, where *E* is the emission, *F* is the radiative forcing and *t* is the time.

## 7. Implications of metric choices on the timing of net-zero emissions

**Figure 2.**
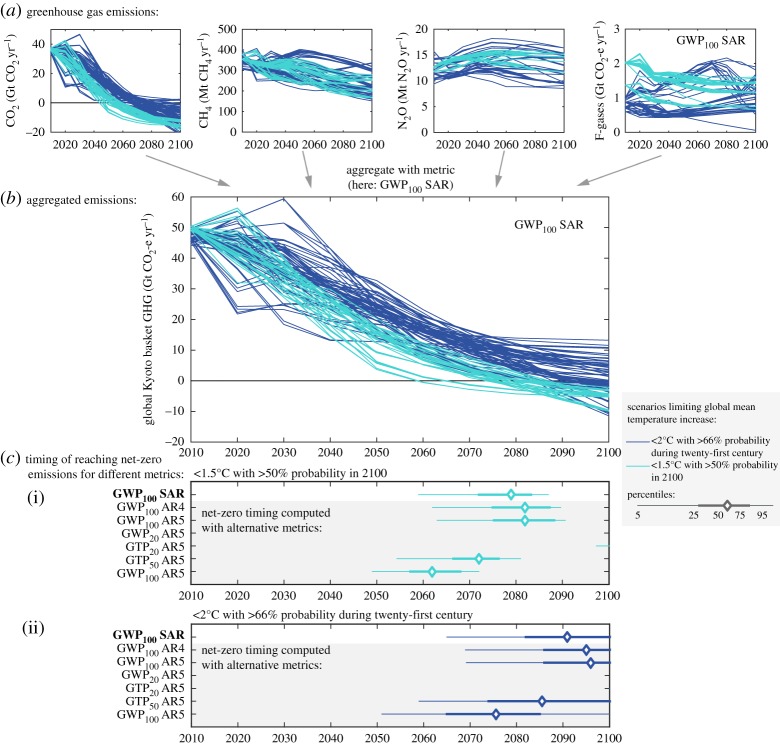
(*a*) Range of permissible GHG emission scenarios for CO_2_, CH_4_, N_2_O and F-gases for the 1.5°C and 2°C goals. (*b*) Example of aggregated emissions paths in terms of CO_2_-equivalent emissions based on GWP_100_ (SAR) indicating timing of net-zero CO_2_-equivalent emissions. (*c*) Timing of net-zero CO_2_-equivalent emissions as a function of metric type and time horizon.

Integrated assessment models (IAMs) are used for calculations of emissions pathways consistent with various levels of global warming. Based on information about physical properties of individual climate forcers (RF, atmospheric lifetimes), available technologies and costs to reduce them, as well as various assumptions about the level of international collaboration or the inclusion of specific options (like land-based options), these models generate internally consistent emissions pathways per forcing agent [25]. The optimal trade-off between emissions of different GHGs is usually calculated based on economic considerations—either by directly evaluating the marginal costs and marginal benefits (in terms of reaching the temperature goal) of reducing an additional unit of emissions for each gas, or by assuming a conversion metric such as the GWP for imposing a uniform price on all GHG emissions. Other forcing agents such as ozone (other than the aforementioned response to methane) and aerosols are usually not accounted for in the economic optimization of climate mitigation measures, and would only adjust to climate policy to the extent its anthropogenic sources respond to GHG control (such as SO_2_ emissions being reduced by phasing out of coal-fired power plants) [26]. For example, figure 2*a* shows emissions pathways consistent with limiting global temperature rise to 1.5°C in 2100 and to 2°C during the entire twenty-first century, with greater than 50% and greater than 66% probability, respectively. Scenarios here have been drawn from the IPCC AR5 Scenario Database, which is hosted by the International Institute for Applied Systems Analysis (IIASA) and available at https://tntcat.iiasa.ac.at/AR5DB/ [27], and from an earlier study on 1.5°C pathways [28]. This set of emission scenarios enables us to show the implications of the remaining emissions by the end of the century and are used for illustrating the effect of choice of metric for the timing of net-zero emissions. Thus, they should not be seen as post-2100 scenarios. The two interpretations of the temperature targets that were introduced above have been chosen because they are commonly used in scientific analyses and policy discussions. The 2°C probability refers to peak warming, while the 1.5°C probability refers to the year 2100. These interpretations do not represent an assessment of the exact meaning of Article 2 of the Paris Agreement.

The individual GHG emission paths (in figure 2*a*) from the IAMs can be aggregated (in figure 2*b*) to ‘CO_2_-equivalent emissions’ by the use of an emission metric. A common choice is GWP_100_ from the IPCC SAR, which was adopted as the standard metric under the Kyoto Protocol. For this reason, the IPCC AR5 used SAR GWP_100_ to aggregate emissions information from IAMs in CO_2_-equivalent terms. As can be seen from figure 2*b*, the CO_2_-equivalent emission paths from different models have different temporal developments, and therefore cross the zero line at various points in time during the second half of the century. The point of reaching net-zero CO_2_-equivalent emissions varies across both models and metrics.

Figure 2*c* shows the impact of using different metrics (i.e. GWP and GTP, and different time horizons) for aggregating the various GHGs to CO_2_-equivalent emissions, focusing on how this affects the *timing* of net-zero emission for our interpretation of 1.5°C (figure 2*c*(i)) and 2°C (figure 2*c*(ii)) scenarios (see electronic supplementary material, table S1 for an overview of metric values).

Figure 2*b,c* shows that choosing a different metric for the aggregation of various GHGs changes their relative contributions and therewith the *perceived* timing of when net-zero GHG emissions are achieved. Policymakers based the text of the Paris Agreement on information that was available to them at the time of drafting. This information, like the IPCC AR5 or the UNEP Emissions Gap Reports, showed CO_2_-equivalent emissions pathways aggregated with the GWP_100_ metric, in most cases with values from SAR or AR4. Changing the CO_2_-equivalence metric from GWP_100_ to any other metric while still keeping the same timing of achieving net-zero GHG emissions could hence potentially introduce an internal inconsistency between paragraph 17 of the Decision associated with the agreement and Article 4 of the agreement itself. It may also be argued that it could introduce inconsistency between Articles 2 and 4—depending on the interpretation of the long-term temperature goal. Any change in the CO_2_-equivalence metric should thus be considered in conjunction with the additional information and context provided in these Articles.

As stated in the Paris Agreement, the time of net-zero emissions for CO_2_-equivalent emissions (based on GWP_100_ from SAR) is in the second half of the century for the two warming targets. For the 1.5°C and 2°C cases, updating the CO_2_ equivalents from SAR to AR4 or AR5 will only shift the timing by a few years. However, for the 2°C median case (not shown) such an update would move the timing of reaching net-zero emissions beyond 2100.

To summarize, when GWP_100_ is used to calculate CO_2_-equivalent emissions from existing pathways, the net-zero timing is not very sensitive to updates from SAR to AR4 or AR5 values. Using GTP_100_ will shift the net-zero timing to earlier periods, due to less weight given to CH_4_. The use of a 20-year time horizon for GWP or GTP would give a net-zero timing after 2100. As residual emissions of CH_4_ persist in all pathways, higher metric values for CH_4_ require larger net negative CO_2_ emissions to establish net-zero CO_2_-equivalent emissions, which happens at a later point in time or may not happen at all in these illustrative scenarios. Correspondingly, a low metric value for CH_4_ (such as GTP_100_) will lead to early net-zero value of the two gases.

**Figure 3.**
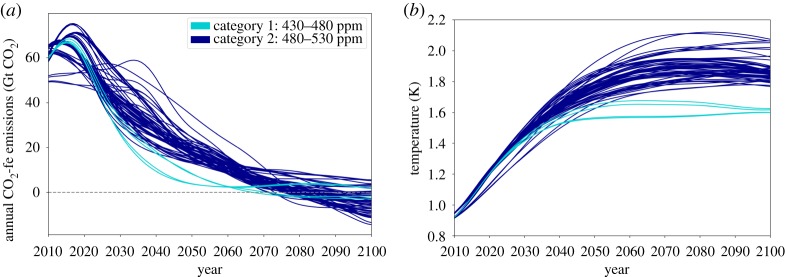
(*a*) Annual CO_2_-fe emissions and (*b*) global mean temperatures for scenarios in the IPCC AR5 Scenario Database. CO_2_-fe emissions have been calculated based on the total anthropogenic forcing in the Scenario Database. Temperatures are taken directly from the Database.

The earlier introduced CO_2_-fe emissions could also be considered as an indicator of balance. Figure 3 shows time series of annual CO_2_-fe emissions and global temperatures for scenarios in the IPCC AR5 Scenario Database, with colours representing the two most ambitious categories for mitigation, as defined in [27]. (Scenarios that treat sulfate and GHGs in an inconsistent manner have been excluded.) The total anthropogenic RF from each scenario has been used to calculate a CO_2_ concentration that equates to that RF [29]. The FAIR model [29] is used to calculate the time profile of CO_2_ emissions required to produce the time profile of CO_2_ concentrations, leading to the CO_2_-fe emissions shown in figure 3*a*. The most ambitious mitigation scenarios (category 1 and 2, light and dark blue) reach net-zero CO_2_-fe emissions around 2070 at the earliest, with some not crossing that threshold until after 2100. (Category 1 scenarios have CO_2_-equivalent concentrations of 430–480 ppm, and category 2 scenarios have 480–530 ppm.) This illustrates the importance of using consistent metrics when considering balance and its timing.

## 8. Effect of metric choice on the implementation of balance and on global temperature

**Figure 4.**
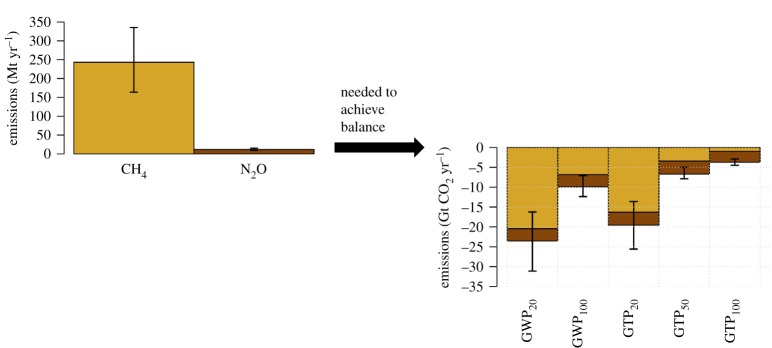
Dependence of negative CO_2_ emissions needed to balance residual emissions of CH_4_ and N_2_O (given as Mt CH_4_ and Mt N_2_O, respectively) on metric choice. For the left-hand panel, the bar height indicates the mean of the distribution of CH_4_ and N_2_O emissions in 2100, with the extent of the error bar marking the 5th–95th percentiles of the distribution. For the right-hand panel the bar heights indicate the mean net negative CO_2_ emissions needed to balance the combinations of 2100 CH_4_ (orange) and N_2_O emissions (brown) present in the scenarios partitioned by metric (*x*-axis). The 5th–95th percentiles of the distribution of the total required net negative CO_2_ emissions are marked by the vertical extent of the bars.

Achieving a ‘balance’ in GHG emissions implies that any difficult-to-abate emissions of CO_2_ and non-CO_2_ gases, expressed in CO_2_ equivalence through a metric of choice, need to be compensated by negative CO_2_ emissions. The choice of metric type and time horizon for calculation of CO_2_-equivalent emissions will affect the magnitude of net negative emissions of CO_2_ needed to produce net-zero CO_2_-equivalent emissions. Figure 4 uses N_2_O and CH_4_ as representative long-lived and short-lived non-CO_2_ gases to demonstrate the effect of metric type and time horizon on the required amount of net negative CO_2_ emissions needed to achieve a net-zero balance in terms of CO_2_-equivalent emissions. GTP_100_ gives substantially lower weight to methane emissions than under GWP_100_, and consequentially smaller amounts of net negative CO_2_ emissions are needed than when considering GWP_100_. In general, metric choice affects the amount of negative CO_2_ emissions needed to balance residual CH_4_ emissions much more than for residual N_2_O emissions. This is due to the fact that N_2_O, like CO_2_, is long-lived, so it can be compared against CO_2_ in a much more like-for-like fashion leading to relatively little variation in values between the different metrics.

**Figure 5.**
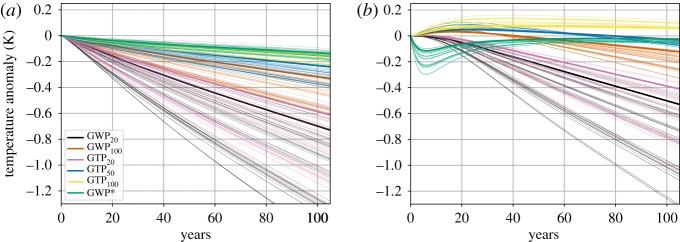
(*a*) Global mean surface temperature response to maintained net-zero balance of CO_2_-equivalent emissions from 2100 for various metrics. Thin lines indicate the range of response for the net-zero balances imposed using constant 2100 CH_4_ and N_2_O emissions from the complete set of 430–480 ppm scenarios, while thick lines indicate the response for the balance imposed from 2100 for the RCP2.6 scenario. (*b*) Calculations are done as perturbations above an RCP2.6 background of emissions with the perturbation implemented from 2100 and onwards. The spread in responses reflects the spread in non-CO_2_ emissions due to scenario and model assumptions.

As the physical consequences of net-zero balance will depend on the choice of metric, it is important to evaluate to what extent differing metrics correspond to differing physical consequences under maintained net-zero CO_2_-equivalent emissions, for example, its global temperature consequences. Figure 5 shows the evolution of global temperature relative to 2100 under the maintenance of net-zero emissions between CH_4_, N_2_O and CO_2_ emissions using different metrics and time horizons. Emissions of CH_4_, N_2_O and (negative) RF due to other (non-CO_2_/CH_4_/N_2_O) climate forcers are held constant at 2100 values after 2100, with removals of CO_2_ imposed in each year to maintain a net-zero balance between the CO_2_, CH_4_ and N_2_O emissions under the range of metrics considered for these highly idealized cases. To assess the global mean temperature response to this balance, we use a simple impulse–response climate model (FAIR) which is calibrated to capture the dependence of the climate response to pulse emissions on the background climate state [29]. Solid lines in figure 5 represent the climate response to net-zero balances imposed from 2100 onwards for the representative concentration pathway RCP2.6 scenario, with the thin lines representing the range of responses in the full set of 430–480 ppm scenarios from the IPCC AR5.

For all the metric choices considered (figure 5*a*), we find a non-zero temperature impact of maintained balance, in part due to (i) the metric-dependent time profiles of CO_2_ removal and its associated cooling, (ii) the continued climate impact of forcing before 2100 and (iii) the cooling effect of additional (negative) non-GHG climate forcing (held constant after 2100) which is not included in the calculation of net-zero balance. Balance based on the 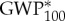 metric comes closest to holding temperatures constant, while the other metrics give stronger cooling effects. As shown by [5], using 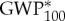, maintaining a constant rate of an emission of CH_4_ becomes equivalent to a zero rate of CO_2_. The other metrics, GWP and GTP, compare, over a finite time horizon, integrated RF from CH_4_ to the integrated RF of CO_2_—which continues to affect the climate on a much longer time scale than CH_4_. The higher the metric value for methane, from 4 for GTP_100_ to 84 for GWP_20_, the larger negative CO_2_ emissions are needed to obtain net-zero CO_2_-equivalent emissions (figure 4), and consequentially larger cooling as a result of net-zero CO_2_-equivalent emissions (figure 5*a*). (It may be noted that this consequence of imposed net-zero balances is hence the opposite to what would occur when CH_4_ emissions are reduced instead of CO_2_—where a long-term warming is the result of trading methane based on GWP equivalence (e.g. [30,31].)

As the climate response to RF is not instantaneous, the temperature responses shown in figure 5*a* are also affected by the forcings from previous emissions, and therefore the post-2100 evolution of warming shown in figure 5*a* is not solely attributable to the effects of the imposed net-zero balance from the emissions shown in figure 4. The isolated temperature effects of the emissions included in any net-zero balance can be evaluated by applying them as a perturbation on top of a background emissions scenario. In figure 5*b,* we use an emissions background of maintained 2100 values of emissions and other forcings in the RCP2.6 scenario and add an additional perturbation of the maintained net-zero balances between 2100 residual CH_4_ and N_2_O emissions (from the individual 430–480 ppm scenarios), and negative CO_2_ emissions according to different metrics, from 2100 onwards. The temperature outcome is then differenced from the standard RCP2.6 case to isolate the effect of the perturbation. In this isolated and idealized case for illustration, the near-term warming of CH_4_ is clearly seen, with warming initially rising under metrics that give a low weight to methane. Conversely, as the GWP* metric balances the sustained CH_4_ emissions with a single pulse removal of CO_2_ in the first year, the cooling effect from this pulse removal is the dominant near-term signal with the net temperature returning towards zero.

Which perspective to use—including effects of previous emissions or the isolated effects of the balanced remaining emissions—is one of the questions that need to be answered to obtain a clear and operational definition of GHG balance.

**Figure 6.**
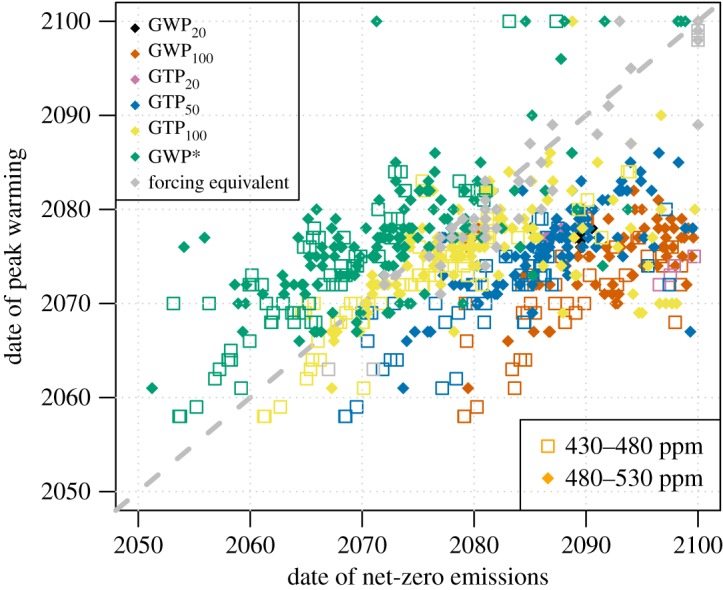
Correlation between time of net-zero emissions and time of peak warming for various metric choices, including CO_2_-fe. Grey dashed line represents the 1 : 1 line. Different colours represent the different metric choices, with open squares denoting the 430–480 ppm scenarios and filled symbols the 480–530 ppm scenarios.

Because the Paris Agreement aims to limit peak warming and it also provides a broad timing for achieving the Article 4 ‘balance’, it is of interest to understand how the timing of achieving net-zero emissions relates to timing of the peak in global mean temperature increase, and how different metrics affect this relationship. Figure 6 shows the timing of net-zero emissions versus the timing of peak warming in the scenarios available in the IPCC AR5 Scenario Database. For a hypothetical case in which only CO_2_ emissions occur, we would expect peak warming to occur very rapidly after the achievement of net-zero emissions [32]. As the CO_2_-forcing equivalent (grey), by construction, achieves a perfect equivalence between other GHGs and CO_2_ in terms of their effect on global temperature, CO_2_-fe shows the closest relationship between the timing of net-zero emissions and the timing of peak warming in figure 6. For the other metrics shown, GWP* (brown) best captures the link between the date of peak warming and the date of net-zero CO_2_-forcing equivalent emissions, with other metrics typically reaching net-zero emissions after peak warming has occurred. (The net-zero timing is calculated using CO_2_, CH_4_ and N_2_O only, so the scatter is also in part associated with the profiles of other forcing agents.) GWP_100_ results in a delay of about 20 years between the timing of peak warming and the timing of net-zero emissions.

Few cases of GWP_20_ and GTP_20_ are seen in figure 6, as with such a high weighting given to methane emissions (84 and 67, respectively) net-zero emissions are not reached before 2100 in the vast majority of scenarios (figure 2). GTP_100_ is again closest to 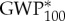 because little weight is given to methane, while in all cases the timing of peak warming is either contemporaneous with the timing of net-zero emissions or occurs later.

An alternative approach to interpret and define balance would be to counterbalance only the long-lived non-CO_2_ GHGs while keeping the emissions of short-lived components constant, as near-stabilized emissions of these components (e.g. CH_4_) would only contribute to small temperature changes after this point (this is, in effect, what net-zero emissions measured by GWP* means). We find that both GWP and GTP applied for CO_2_-equivalent emissions of N_2_O will give good compensation of the temperature effect and that the variance of the N_2_O-induced warming between metric choices is small (electronic supplementary material, figure S1a and S1b). Note that despite being denoted as zero CO_2_-equivalent emission based on GWP*, a constant and ongoing high level of SLCF emissions may still represent a significant contribution to warming and a mitigation opportunity.

## 9. Discussion and conclusion

Article 4 of the Paris Agreement stipulates that in order to achieve the long-term temperature goal of the Paris Agreement, countries aim to achieve a ‘balance between anthropogenic emissions by sources and removals by sinks'. Based on available evidence in the UNFCCC process, it seems clear that the word ‘anthropogenic’ applies to both ‘emissions by sources' and ‘removal by sinks’. Issues related to the scope of ‘anthropogenic’ remain, for instance, when natural sinks are indirectly enhanced, weakened or reversed by anthropogenic perturbations. Development and application of robust and transparent reporting methods will be essential, including definitions of system boundaries and clarification of time scales. In particular, the issue of *permanence* of the CO_2_ removal process under consideration is important, and applies to both industrial and ecosystem-based CO_2_ removals. There is a range of time scales for carbon removals—from short time scales (soil carbon that is later perturbed), over long time scales (soil carbon that is not perturbed for decades, or surface ocean), further to very long time scales (deep ocean, geological storage with leakage), and, finally, almost infinite (geological storage with no leakage, mineralized carbon). It may be argued that if a storage is non-permanent, it has to be discounted and replaced later by another form of storage. Despite its limitation, non-permanent storage could play a role in the near term.

A balance between anthropogenic sinks and sources of GHG emissions can be defined in several ways. From a scientific point of view, these various options translate into a most appropriate choice of metric for weighting emissions. The Paris decision highlights the importance of using common metrics as assessed by the IPCC (cf. paragraph 31 of Decision 1/CP.21). However, more specific guidelines might be developed. As demonstrated, balancing a steady rate of methane and other short-lived emissions with active removal of CO_2_, the option of achieving net-zero emissions under conventional emissions reporting procedures (i.e. based on GWP_100_) results in a steady decline of global temperatures.

Clarifying the scope of ‘removals by sinks’ for different GHGs is ultimately a matter for the UNFCCC; our role here is to point out the implications of different interpretations. One option that could be consistent with the context of Article 4 would be to consider a removal to be any anthropogenic activity that results in a permanent (or at least verifiably multi-century) reduction in the concentration of a GHG in the atmosphere. For CO_2_, this means active capture and sequestration. For methane, this could mean the permanent enhancement of a natural sink, in this case induced by the methane emissions themselves. Simply actively removing a fixed quantity of methane is of less relevance, because concentrations would recover within a few decades after the removal activity ends, unlike the removal of CO_2_, for which the benefits are effectively permanent. These potential interpretations of ‘removals’ point to the need for clarification by policymakers. If deemed appropriate, such interpretation would also point to the use of a metric like GWP* relating rates of emission of SLCFs with cumulative emissions of CO_2_ in the definition of ‘net-zero emissions’. It is worth noting that a constant and ongoing high level of SLCF emission may still represent an important contribution to warming and a mitigation opportunity even if it is denoted as zero CO_2_-equivalent emission based on GWP*.

Furthermore, the Paris text requests countries to include all categories of emissions and removals in their nationally determined contributions and GHG inventories. However, the Paris Agreement itself is not specific on which components are included in the balance calculations of CO_2_-equivalent emissions. From the Agreement text it is clear that aerosols are not included. Based on current practice under the UNFCCC, it seems reasonable to assume that any balance assessment will be applied to the Kyoto basket of GHGs, although this has yet to be specified. SRM by injection of sulfate aerosols into the atmosphere is clearly excluded from the balance considerations as covered in the Paris Agreement.

From a geophysical perspective, the balance between sources and sinks is needed at a global, not national, level. However, there are significant differences across countries in anticipated residual emissions and potentials for negative CO_2_ emissions. To achieve net-zero CO_2_-equivalent emissions and balance at a global level, mechanisms will be needed for strong international coordination. In this context, Article 4 already indicates that emissions reductions and the achievement of a balance between sinks and sources should be carried out ‘on the basis of equity, and in the context of sustainable development and efforts to eradicate poverty’. This results in some key questions, some of which are scientific and some of which are political in nature. For example, understanding synergies and trade-offs of specific mitigation measures with the achievement of the Sustainable Development Goals becomes increasingly important and cannot be simply related to global temperature change. At the same time, political and ethical issues related to who should pay for creating and maintaining sinks, or who would be responsible for the sinks’ effectiveness and durability over time, have to be discussed and resolved through a political dialogue.

In conclusion, we have clarified how from a scientific perspective the ‘balance’ referred to in Article 4 of the Paris Agreement may be applied to anthropogenic sources and anthropogenic sinks. We have shown how the timing of nominal ‘net-zero’ CO_2_-equivalent emissions depends on choice of metric. Changing from GWP_100_ to any other metric while still keeping the same timing of achieving net-zero GHG emissions could introduce an inconsistency between paragraph 17 of the Paris decision and Articles 2 and 4 of the Paris Agreement itself. Depending on the interpretation of the long-term temperature goal described in Article 2, some metrics might be better suited to implement the balance of Article 4. In particular, we clarified that the way in which a balance is achieved and maintained influences the anticipated temperature outcome over time. For example, achieving and maintaining net-zero CO_2_-equivalent emissions calculated with GWP_100_—adopted for the implementation of the Kyoto Protocol and in UNFCCC reporting—would result in a peak and decline in global temperature and a cooling effect, with the rate of cooling dependent on the contribution of SLCFs to the overall CO_2_-equivalent emissions. Adopting a different usage of this metric, here denoted by GWP*, would result in global temperatures remaining approximately constant once net-zero CO_2_-equivalent emissions are achieved and maintained. Policymakers should be aware of these particularities and determine which metric is most appropriate in the context of the goals of the Paris Agreement.

## Supplementary Material

Supplementary Material

## Footnotes

1 The French version states: ‘de façon à parvenir à un équilibre entre les émissions anthropiques par les sources et les absorptions anthropiques par les puits de gaz à effet de serre au cours de la deuxième moitié du siècle’. The Spanish version states: ‘para alcanzar un equilibrio entre las emisiones antropógenas por las fuentes y la absorción antropógena por los sumideros en la segunda mitad del siglo’. The Russian, Chinese and Arabic versions mirror the English wording.

## Data accessibility

This article has no additional data. Scenarios are drawn from the IPCC AR5 Scenario Database, hosted by IIASA at https://tntcat.iiasa.ac.at/AR5DB/. Electronic supplementary material is available online at rs.figshare.com.
