# Supplementary material for: Implications of possible interpretations of ‘greenhouse gas balance’ in the Paris Agreement
Source: Philos Trans A Math Phys Eng Sci. 2018 Apr 2;376(2119):20160445. doi: 10.1098/rsta.2016.0445 (PMC5897819; doi:10.1098/rsta.2016.0445)
Supplement: Supplementary Material [file rsta20160445supp1.docx]

**Supplementary Material**

**Table S1: GWP and GTP values for CH_4_ and N_2_O from various IPCC reports.**

|  | **GWP_20__CH_4_** | **GWP_100__CH_4_** | **GWP_100__N_2_O** | **GTP_20__CH_4_** | **GTP_50__CH_4_** | **GTP_100__CH_4_** | **GTP_20__N_2_O** | **GTP_50__ N_2_O** | **GTP_100__ N_2_O** |
| --- | --- | --- | --- | --- | --- | --- | --- | --- | --- |
| SAR | 56 | 21 | 310 | - | - | - | - | - | - |
| TAR | 62 | 23 | 296 | - | - | - | - | - | - |
| AR4 | 72 | 25 | 298 | - | - | - | - | - | - |
| AR5 | 84 | 28 | 265 | 67 | 14 | 4 | 277 | 282 | 234 |


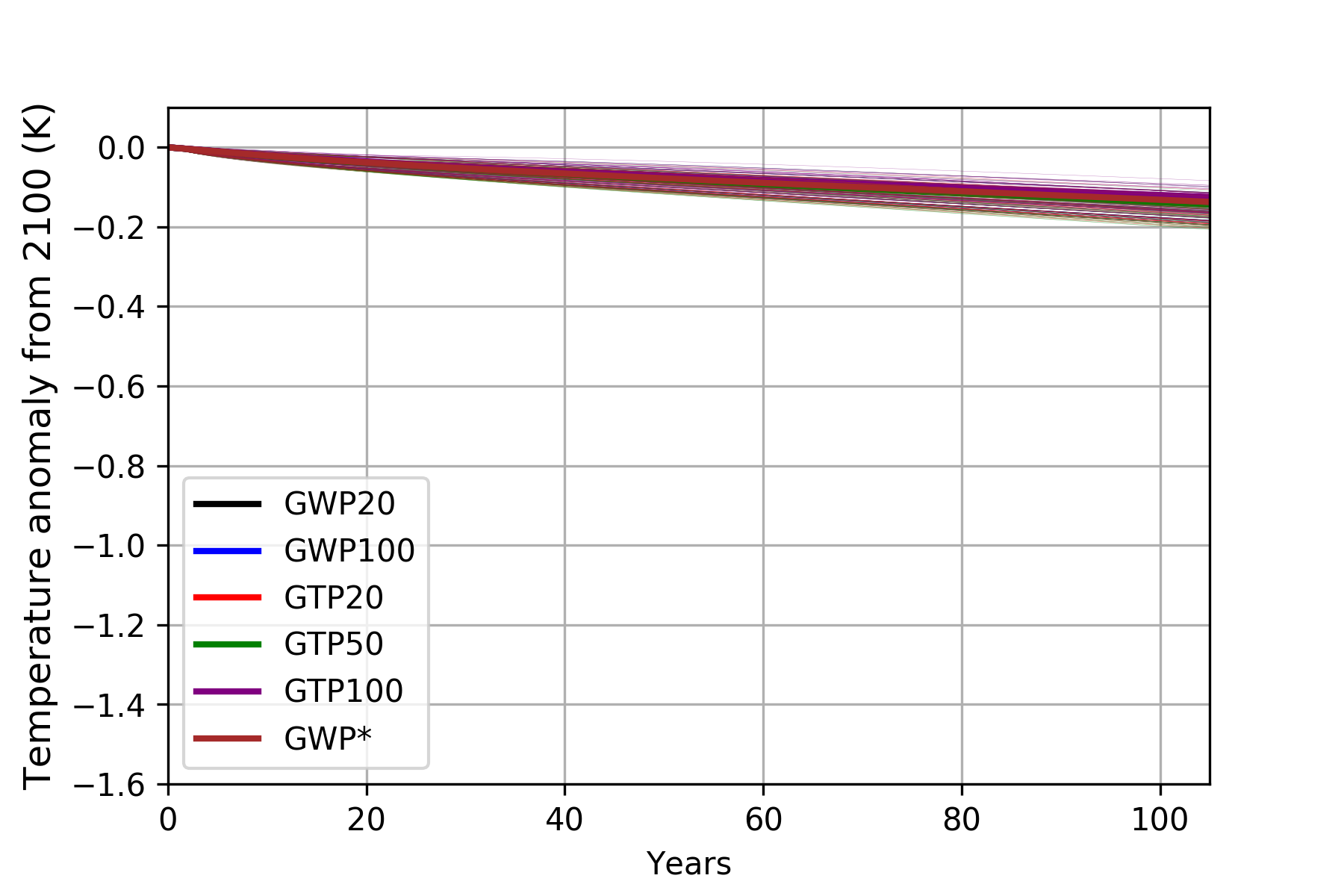

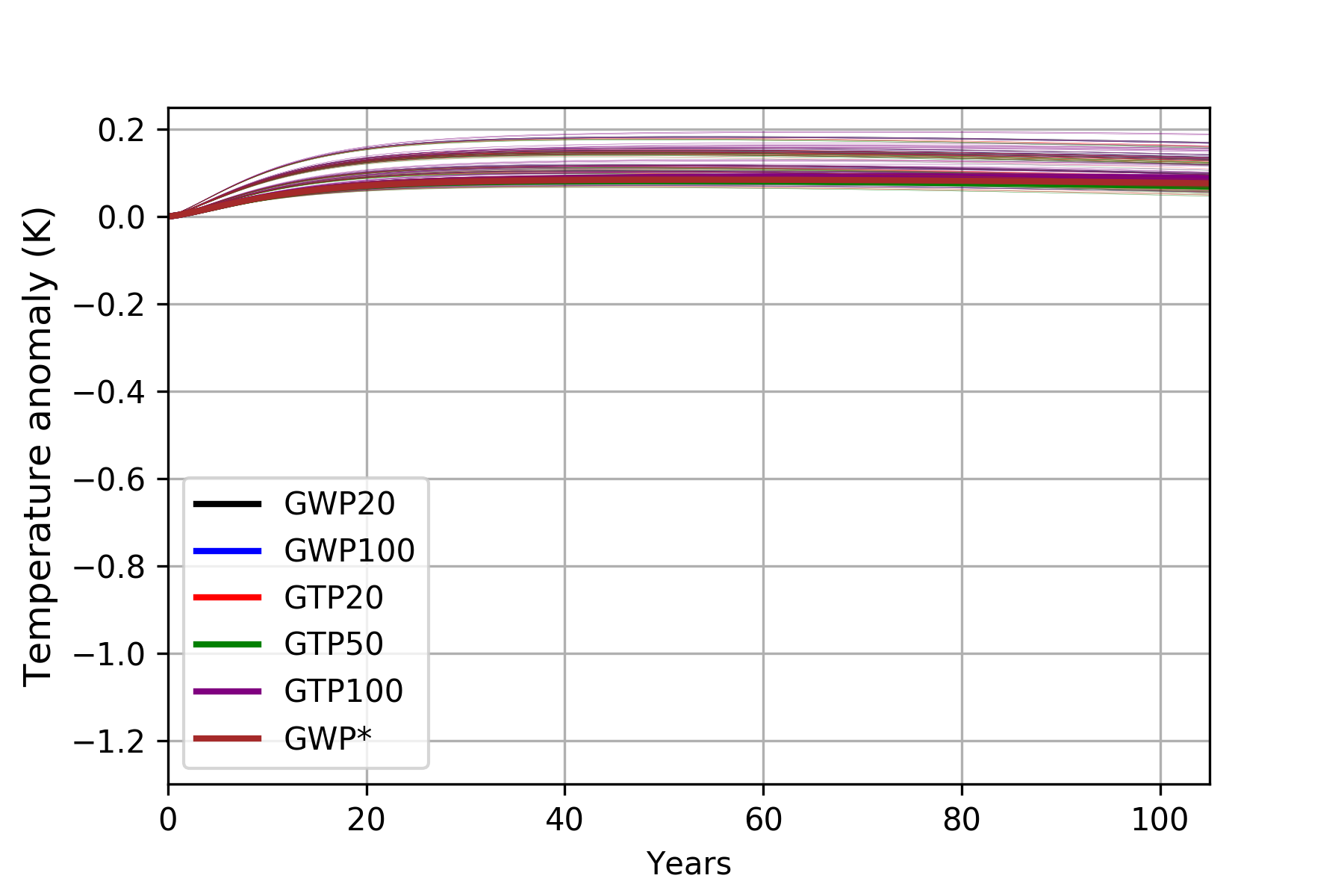


**Figure S1**. As figure 5a and 5b, but with constant residual CH_4_ emissions not included in the calculation of off-setting net negative CO_2_ emissions to achieve net-zero balance.
